# Developing Clinically Relevant Dissolution Specifications for Oral Drug Products—Industrial and Regulatory Perspectives

**DOI:** 10.3390/pharmaceutics12010019

**Published:** 2019-12-23

**Authors:** Mark McAllister, Talia Flanagan, Karin Boon, Xavier Pepin, Christophe Tistaert, Masoud Jamei, Andreas Abend, Evangelos Kotzagiorgis, Claire Mackie

**Affiliations:** 1 Pfizer PGRD, Sandwich CT13 9NJ, UK; Mark.McAllister@pfizer.com; 2 AstraZeneca UK Limited, Macclesfield SK10 2NA, UK; Talia.Flanagan@ucb.com (T.F.); Xavier.Pepin@astrazeneca.com (X.P.); 3 UCB Pharma SA, 1420 Braine l’Alleud, Belgium; 4 Licensing Division, MHRA, London E14 4PU, UK; Karin.Boon@mhra.gov.uk; 5 Janssen Research and Development, 2340 Beerse, Belgium; CTISTAER@its.jnj.com; 6 Certara UK, Simcyp Division, Sheffield S1 2BJ, UK; Masoud.Jamei@certara.com; 7 Merck & Co., Inc., West Point, PA 19486, USA; andreas_abend@merck.com; 8 European Medicines Agency, 1083 HS Amsterdam, The Netherlands; Evangelos.Kotzagiorgis@ema.europa.eu

**Keywords:** oral, drug products, clinically relevant dissolution specifications, PBBM, product performance, biorelevant dissolution

## Abstract

A meeting that was organized by the Academy of Pharmaceutical Sciences Biopharmaceutics and Regulatory Sciences focus groups focused on the challenges of Developing Clinically Relevant Dissolution Specifications (CRDS) for Oral Drug Products. Industrial Scientists that were involved in product development shared their experiences with in vitro dissolution and in silico modeling approaches to establish clinically relevant dissolution specifications. The regulators shared their perspectives on the acceptability of these different strategies for the development of acceptable specifications. The meeting also reviewed several collaborative initiatives that were relevant to regulatory biopharmaceutics. Following the scientific presentations, a roundtable session provided an opportunity for delegates to discuss the information that was shared during the presentations, debate key questions, and propose strategies to make progress in this critical area of regulatory biopharmaceutics. It was evident from the presentations and subsequent discussions that progress continues to be made with approaches to establish robust CRDS. Further dialogue between industry and regulatory agencies greatly assisted future developments and key areas for focused discussions on CRDS were identified.

## 1. Introduction

In November 2017, a one-day meeting titled ‘*Developing Clinically Relevant Dissolution Specifications for Oral Drug Products—Industrial and Regulatory Perspectives*’ was convened by the APS Biopharmaceutics and Regulatory Sciences focus groups. Developing and registering clinically relevant dissolution specifications has been the subject of much discussion and debate for formulation, analytical, and regulatory scientists for several years [1], and recent workshops that were focused on this topic suggest that much more needs to be done to have an agreed scientific framework to routinely establish such specifications for oral drug products [2,3,4].

Dissolution testing is often used to establish the impact of formulation and manufacturing process changes during oral drug product development. Effective strategies for dissolution method development and establishing relevant acceptance criteria are essential in ensuring drug product quality. Currently, during late-stage drug product development, the formulation and process parameters are varied to produce so-called process and formulation variants that are used to test the discriminatory ability of a dissolution method to detect changes in oral drug product quality. However, the adopted approaches create formulations variants that can be considered to be clinically relevant or meaningful in the context of composition or process variation can be diverse and regulatory guidelines for this type of experimentation are not yet clearly defined [5]. Recent advances with in silico mechanistic absorption/physiologically based biopharmaceutics modelling (PBBM) have increased our understanding of in vivo oral drug product performance and they are being increasingly used to establish clinically relevant specifications [6]. This has highlighted the requirement for clinically relevant and/or biorelevant dissolution data to support model development and the need to increase the general level of understanding and confidence in the modelling of oral drug product performance for this purpose. This meeting was intended to contribute to the longer-term goal of the development of a regulatory framework to encompass this rapidly growing field. Industrial scientists that were involved in product development were invited to share their experiences with a range of in vitro dissolution and in silico modelling approaches to establish robust clinically relevant dissolution specifications to achieve this aim. It was also important to provide an opportunity for regulatory scientists who have had experience in reviewing applications that contained CRDS to share their perspectives on the acceptability of different strategies for the development of acceptable specifications. The meeting program also reviewed several collaborative initiatives that were relevant to regulatory biopharmaceutics, such as ICH M9 (BCS-Based Biowaivers) [7] and the IMI OrBiTo research program (www.orbitoproject.eu). After the scientific presentations, the meeting concluded with a roundtable session that provided delegates with an opportunity to discuss the information shared and to debate the options to make progress in this critical area of regulatory biopharmaceutics.

## 2. Summary of Podium Presentations

### 2.1. Clinically Relevant Specifications: Connecting Product Performance to the Patient—Talia Flanagan (AstraZeneca)

The principles and benefits of clinically relevant specifications are now relatively well established. These include the ability to optimize the manufacturing process and evaluate changes that are based on in vivo performance, enhanced security of product supply, and improved assurance of the clinical quality of product supplied to patients. However, this area continues to evolve, as industry and regulators learn from working with CRDS in the real world and seek to apply the latest tools and techniques to link in vitro product performance to the patient. An open dialogue is needed between industry and regulators on how the link between in vitro and in vivo performance has been established during product development and how this understanding evolves during the product lifecycle to realize the full benefits of CRDS.

The Biopharmaceutics Classification System (BCS) provides a well-established framework for assessing the biopharmaceutics risk of immediate release drug products, based on properties of the Active Pharmaceutical Ingredient (API) and drug product [7,8,9,10]. The compounds are classified into one of four groups, according to their solubility and permeability properties. Compounds in BCS Class 1 (high solubility, high permeability) and BCS Class 3 (high solubility, low permeability) are eligible for biowaivers, i.e., the requirement for an in vivo bioequivalence study is waived if certain requirements are met [7,8,9,10]. For the development of CRDS for these compounds, the BCS effectively acts as an accepted body of ‘prior knowledge’, as the ability of dissolution in simple aqueous buffers across the physiological pH range to ensure in vivo bioequivalence is already well established. This can form the basis of CRDS for BCS Class 1 and 3 compounds, unless additional data are generated to justify the further widening of these limits for a specific drug product.

An ICH guideline (ICH M9) on BCS-based biowaivers has recently been finalised 7]. This guideline provides globally harmonised recommendations for supporting the BCS classification of medicinal products, and data that are needed to support BCS-based biowaivers, including in vitro dissolution tests and acceptance criteria.

BCS Classes 2 and 4 span a much broader range of compound properties and, hence, a wide spectrum of biopharmaceutics risk. Therefore, the relationship between in vitro dissolution and in vivo performance for Class 2 and 4 compounds needs to be explored and understood on a compound-specific basis [11,12]. This will involve evaluation of product-specific clinical data to evaluate the relationship between in vivo performance and in vitro dissolution; in many cases, a clinical relative bioavailability study is performed with the specific aim of supporting development of CRDS. Such studies typically involve the generation of Pharmacokinetics (PK) profiles for drug product process and formulation variants with different in vitro dissolution performance. This approach is described, in detail, in a recent cross-industry White Paper on approaches for development of CRDS, which is later discussed in this manuscript [13].

**Figure 1:**
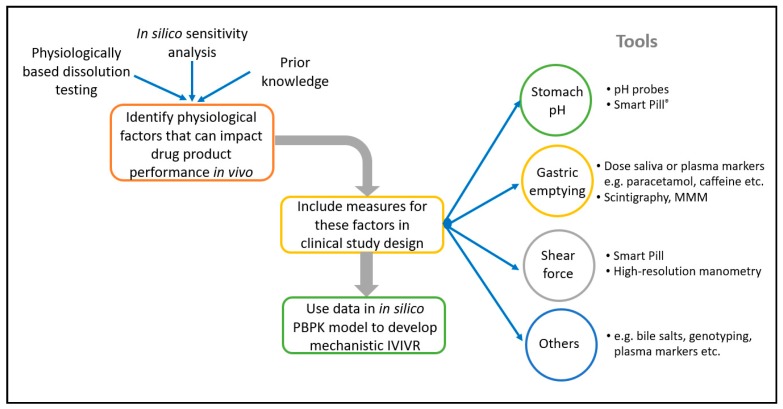
Opportunities for characterisation of the gastrointestinal environment in individual subjects in formulation studies.

The use of in silico PBBM modelling to develop CRDS is becoming more widespread, and several examples of this have been presented by both Industry and Regulators [6,14,15,16]. This approach offers enhanced mechanistic insight into the factors that influence absorption and the interplay between in vitro and in vivo performance. Several examples of the application of in silico PBBM modelling to develop CRDS are described later in this manuscript. An interesting further evolution of this approach is to incorporate elements into clinical studies with the specific aim of feeding the in-silico model. For example, monitoring the gastrointestinal environment in individual patients during formulation PK studies (e.g., gastric emptying time, pH) can lead to an enhanced mechanistic understanding of the formulation performance. This enables the development of improved in silico absorption models by enabling the ‘noise’ from physiological variability to be separated from true product performance [6,17]. A number of established tools and techniques can be incorporated into clinical PK studies to characterise the in vivo environment for the dissolution in individual patients, and underpin the development of in silico models to support CRDS, including, for example, Smart Pills^®^, salivary measurement of paracetamol pharmacokinetics to characterise gastric emptying, high resolution manometry, and magnetic marker monitoring [18,19,20,21]. The linking tools or techniques to apply can be selected based upon the specific properties of your API and drug product, to target the aspects of physiological variability that are most likely to impact drug product performance. Figure 1 illustrates this.

Closer integration of efficacy and safety data into CRDS is another area for future development of CRDS, i.e., moving beyond setting specifications that assure pharmacokinetic similarity to specifications that are based on therapeutic outcomes. Applying the Biopharmaceutics Risk Assessment Roadmap (BioRAM) approach offers an opportunity for achieving this. BioRAM is a strategy and framework for patient-centric product development [22,23]. BioRAM is a systems approach, which encompasses and integrates formulation technology, manufacturing, analytical, and biopharmaceutics aspects of pharmaceutical development with an understanding of pharmacology, clinical endpoints, and patient needs, while using a Roadmap and Scoring Grid. A key element of BioRAM is the development of robust links between drug product performance and the intended clinical outcome, i.e., clinical relevance. While using the BioRAM approach, building understanding of the links between drug delivery and dosage form design with the intended therapy and the targeted patient population is a focus of development from the start. The models are built linking drug product characteristics and in vitro and clinical performance, which can be used to predict the impact of changes and establish clinically relevant specifications in the QTPP. Designing and developing the drug product with the patient in mind means that clinical relevance is built in from the start, and the release methods and specifications represent the culmination of this understanding.

### 2.2. Dissolution: The Regulatory Perspective—Karin Boon, MHRA

As part of product control, dissolution testing is one of the main routine tests for demonstrating that each batch of product manufactured performs the same way in the patients as the batches tested in the clinical trial studies. Therefore, the development of the dissolution method, justification of the dissolution limits and the dissolution data, throughout the method, formulation and process development, for the initial application, and for further lifecycle changes play a central role during regulatory assessment.

For method development, the principles of the PhEur [24] and a recent CHMP reflection paper [5] should be taken into account. The method chosen should be robust, reproducible, and discriminatory. Product specific parameters, such as media, stirring rate, sampling time, and the use of sinkers should be specified and justified based on data. The discriminatory nature should be understood as the ability of the method to differentiate between batches with respect to critical process parameters and/or critical material attributes that may have an impact on the bioavailability of the product [25]; data to support this could, for example, include variations in the stirring speed, particle size distribution, excipient proportions, or stressed samples. More drastic changes, such as the omission of excipients or changes in the manufacturing method are not reflective of expected process variations and should, therefore, not be used. The discriminatory nature of the method should be shown for the timepoint that is specified in the finished product specification, as routine Quality Control (QC) testing will be based on a single time point, rather than on multiple point dissolution profiles. It might be difficult to demonstrate for BCS class I and III drug substances. The suitability of the approved method should be considered in the case of variations during lifecycle management.

Target dissolution profiles will be determined by the desired release and profiles of the clinical batches for new products or by the dissolution characteristics of the reference medicinal product for generic applications. Correspondingly, finished product release and shelf life specification limits will be set:

For originator products: based on the clinical trial batches, possibly supported by an IVIVC; and,

For generic products: based on the batch(es) shown to be bioequivalent to the originator product. For immediate release solid oral dosage forms, the recent CHMP reflection paper [5] suggests that the mean dissolution of 12 units minus 10%, rounded to the nearest 5%, should be used as the Q value and set as Q = 75%, 80%, or 85% in 15/30/45 min. In the case of BCS biowaivers, ≥80% Q in 15 min (class I or III) or 30 min (class I) should be applied.

For prolonged and modified release products similar considerations apply, but multiple point specification limits that reflect the specific release profiles should be set in line with the relevant guidance [24,25,26,27].

The PhEur [24] lists three levels of testing; compliance with level 2 is expected during routine manufacture. This means that, for conventional release products, the average of 12 units (6 + 6) is equal to or greater than Q, and no unit is less than Q—15 %. Separate requirements for prolonged and modified release products are also detailed in the PhEur.

Data provided in support of bioequivalence studies should include dissolution profiles of 12 units generated in three different buffers (normally pH 1.2, 4.5, and 6.8; no surfactants) and the QC media, obtained with batches of test and reference products that were used in the bioequivalence study [8]. A similarity assessment between the test and reference profiles in line with the guideline should be performed, e.g., f2 > 50 or ≥ 85% dissolved in 15 min. Similar data should be provided for full BCS biowaivers and strength biowaivers, although additional conditions, as listed in the guideline, should be considered. For BCS biowaivers, the ICH workplan for ICH M9 [10] should be noted. This aims to harmonise the issues relating to the BCS classification of a drug substance (e.g., highest dose versus highest strength in the solubility assessment) as well as the supportive waiver data that are needed (e.g., dissolution test conditions, critical excipients that may influence the rate and/or extent of absorption). The current differences between regions mean that multiple data sets may be required for global products, in support of a waiver or that, in rare cases, even the BCS classification of a drug substance might differ.

The PhEur [26] describes compositions for both SIF and SGF that are considered as more biorelevant media. While the use of enzymes is acceptable, even encouraged, for modified release products [27] if their type and concentration are justified, as this might improve the correlation to in vivo data and might detect potential food effects, the CHMP guideline on the investigation of bioequivalence [8] stipulates that SIF and SGF without enzymes only are currently acceptable for BCS biowaivers for immediate release products.

### 2.3. In Silico PBBM Modelling in Support of Drug Product Dissolution and Drug Substance Particle Size Specifications—Xavier Pepin, AstraZeneca

Three industrial case-studies were presented, where physiological based biopharmaceutics modelling (PBBM) was used in the regulatory context to support changes to existing dissolution specifications, definition of safe space for product dissolution and drug substance particle size or level 3 site change for a modified release formulation.

For Priadel^®^ 200 mg immediate release tablets, failing to meet dissolution specifications at the end of product shelf life led to batch destruction and significant costs associated to warehouse management for the manufacturing plant. Dissolution specifications were changed post-approval by shifting them towards earlier time points. PBBM was used to justify this change. The model was built and verified on historical PK data for several formulations of lithium salts showing dissolution rates that covered the space of Priadel^®^ 200 mg. The dissolution rate was input in GastroPlus™ model while using a Weibull function. The model was then used to test virtual batches that would dissolve following the current and proposed new dissolution specifications. The exposure ratios that were obtained from these simulations demonstrated bioequivalence in the proposed space. UK and Irish authorities accepted the post-approval change in Spring 2012.

Triapin^®^ is a fixed dose combination of Ramipril immediate and felodipine extended release dosed at 2.5 mg or 5 mg for both actives. During a level 3 site transfer, supportive dissolution testing was performed while using the proposed QC method and showed in vitro equivalence between test and reference batches. In parallel, PBBM was conducted to demonstrate that the QC dissolution method is clinically relevant. The model was built from intra-venous human PK data and in vitro CYP3A4 kinetic data for predicting systemic and first pass gut extraction. In vitro dissolution of extended release formulation variants of felodipine were input in the model with the Weibull function. An equivalent level A correlation for PK profiles and PK parameters for the tablet variants comprising the commercial tablet release rate was demonstrated and the QC dissolution method was shown to be clinically relevant.

**Figure 2:**
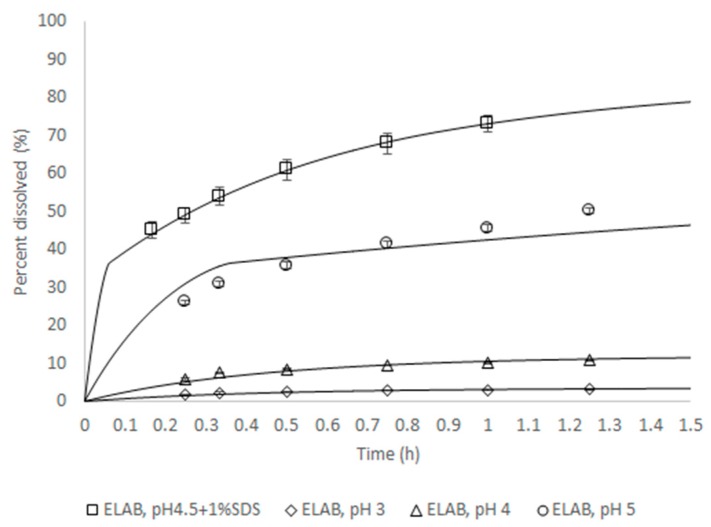
In vitro dissolution rate for batch ELAB fitted using the Quality Control (QC) method or predicted for all other conditions. The lines are predictions and symbols measurements.

For Zurampic^®^ 200 mg, PBBM was conducted during the review period of the NDA submission to the FDA [6] to support the proposed dissolution specification by defining the acceptable safe space for dissolution. The model was built from a population of 10 individual subjects for which intra-venous and oral data were available. The model was verified by its ability to reproduce the outcome of independent clinical trial where a batch of product was shown not to be bioequivalent to the clinical reference. The approach to integrate dissolution data was mechanistic, in that a product particle size distribution (P-PSD) was fitted to the in vitro dissolution profile and then used as an input to the model. This P-PSD allowed for explaining the dissolution rates in other medium compositions (Figure 2).

**Figure 3:**
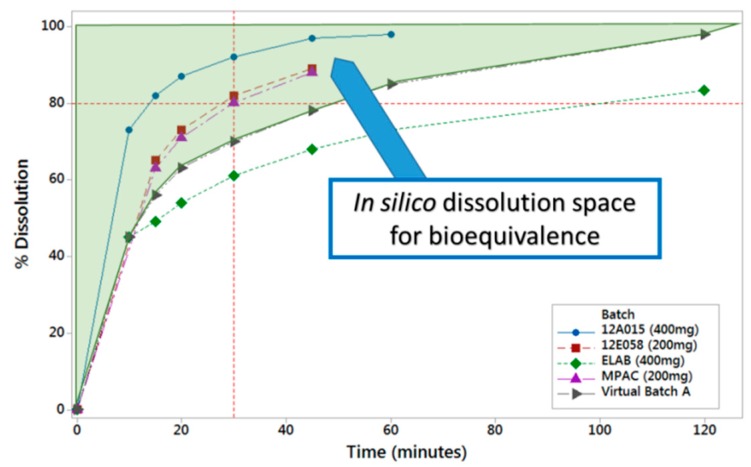
In vitro dissolution space where lesinurad products are predicted to be bioequivalent based on in silico absorption modelling.

PBBM was applied through running virtual clinical trials while using reference and virtual batches to determine the in vitro dissolution space where products are anticipated to be bioequivalent Figure 3). The same model was also used to show that the proposed drug substance particle size specifications were acceptable.

### 2.4. Setting Clinically Relevant Specifications on Polymorphic Purity in a Post-Approval Environment: A Case Study—Christophe Tistaert, Janssen

During the experiments for continuous process improvements of JNJ-1, a new crystal form has been isolated from the drug substance crude crystallization step. The new form appeared to be the thermodynamically favoured form in the process conditions. The compound is classified as a BCS class 4 compound, which is characterized by poor solubility and intermediate permeability, a biopharmaceutical risk assessment was necessary to elucidate the potential differences between both forms that may influence the in vivo performance, and thus the bioavailability of the compound.

In a first step, the physico-chemical characteristics of both API forms were compared in biorelevant media. Table 1 provides an overview of the solubility data and intrinsic dissolution properties. For both API forms, the compound is practically insoluble in water, irrespective of pH, but it undergoes substantial solubilization in the presence of bile. Furthermore, a tenfold increase in solubility and intrinsic dissolution rate were observed when comparing the biorelevant intestinal media and resulted in a tenfold increase between the fasted and fed state simulating intestinal fluids. The absence of any effect of a high-fat meal on the absorption of JNJ-1 indicates that such differences in solubility will not likely affect the intestinal absorption of JNJ-1 at the clinically relevant dose strengths. However, as the solubility of the newly isolated form is about 40% lower when compared to canagliflozin hemihydrate, the bioavailability in the fasted state conditions remains uncertain.

**Figure 4:**
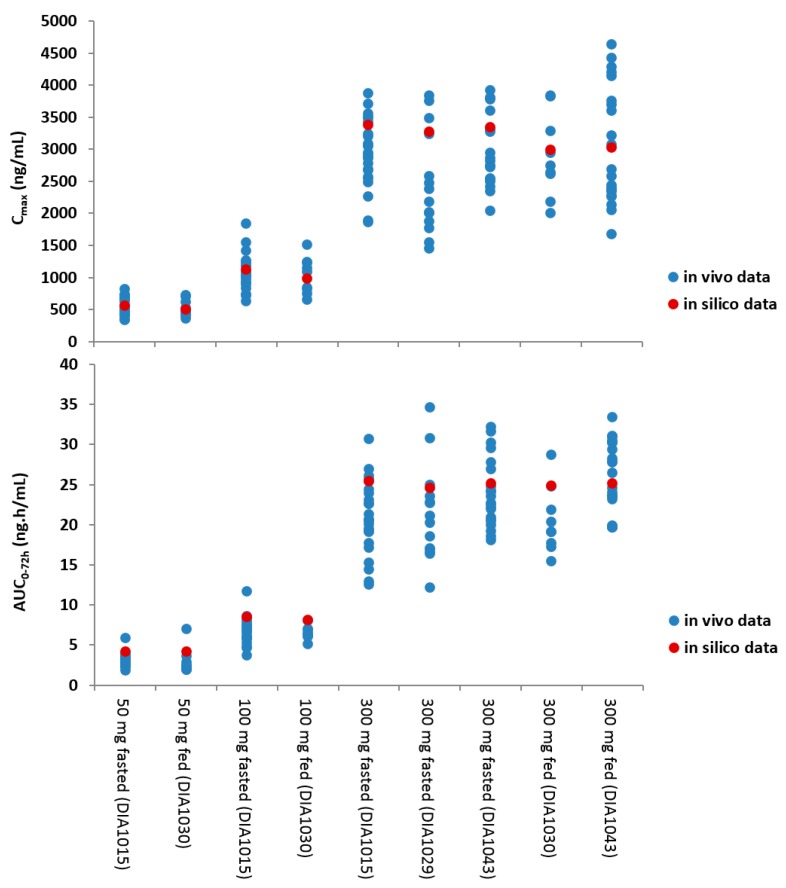
Visualization of the in silico versus the in vivo pharmacokinetics (PK) parameters at different dose levels and in fasted and fed conditions. The blue dots represent the in vivo data, the red dot represents the in silico mean prediction.

The integration of the biopharmaceutical parameters relevant for drug absorption in a more physiological context further expanded the risk assessment. For this, a PBBM model was developed and validated for JNJ-1 while using GastroPlus™ (version 8.5). Based on the available physicochemical properties, in vitro biopharmaceutical experimentation, biorelevant dissolution data, and clinical PK parameters, the model was able to simulate the in vivo observed clinical PK data. The in vitro dissolution based mechanistic absorption model resulted in accurate predictions of the clinical observations at different dose levels in the fasted and fed conditions (Figure 4). The model was able to confirm the demonstrated absence of clinically relevant effects on the oral bioavailability within the particle size specification settings and it was able to successfully simulate the outcome of the food effect study.

**Figure 5:**
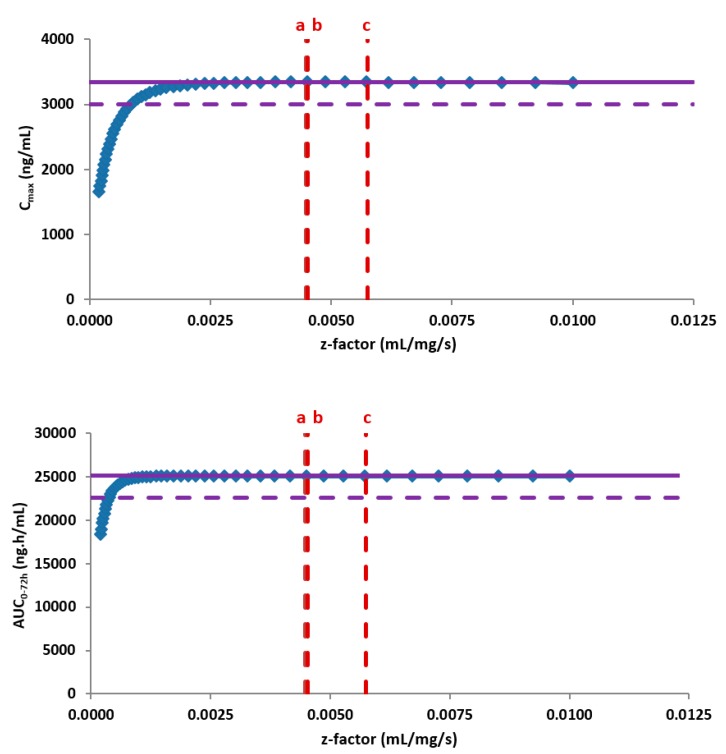
Z-factor parameter sensitivity analysis demonstrating the insensitivity of the PK parameters in fasted conditions to the dissolution rate of the available Active Pharmaceutical Ingredient (API) batches from the new crystal form. The purple solid line represents the clinical exposure of JNJ-1 (reference data), the purple dotted line corresponds to a 10% difference in exposure, the blue dots represent the outcome of the z-factor parameter sensitivity analysis and the red dotted lines correspond to the z-factor calculated from the available API batches of the new crystal form.

Subsequently, the PBBM model was used as an in silico risk assessment evaluating the impact on the bioavailability of the new form after oral administration of the highest clinical dose under the most discriminative conditions. For this, the in vitro dissolution based PBBM model was combined with the biopharmaceutical parameters that were specific for the new crystal form. The modelling and simulations outcome did not indicate any changes in the oral bioavailability when comparing both forms based on an extensive parameter sensitivity analysis. While no risks towards the safety of the patients were identified due to permeation rate limitations, a negative effect on the oral bioavailability and absorption rate would require a significantly slower physiology based in vitro dissolution rate, well below the data from available API batches (Figure 5).

At a later point in time, a confirmatory clinical study performed bridging in modelling and simulations outcome with the in vivo data. The study was a single dose, open-label, randomized four-way crossover pivotal study in healthy adult subject under fasted conditions comparing the reference formulations with three test formulations containing 10%, 50%, and 100% of the new crystal form, respectively, at the highest clinical dose. All of the formulations were found to be bioequivalent, which confirmed the outcome of the in vitro and in silico biopharmaceutical risk assessment.

### 2.5. Integrating In Vitro and In Silico Modelling—OrBiTo Experiences—Masoud Jamei, Simcyp

In vitro dissolution testing is routinely conducted at various stages of drug development for various purposes. A range of apparatuses is used for carrying dissolution testing, and each is intended for specific purposes. The outcome of these tests is often represented as percentages of dissolution of initial dose over time. Such data provide valuable information regarding both the API and the formulation, so those can be used to get more insight in the drug and drug products behaviour in different environments.

The dissolution testing data can be used to anticipate/predict drug products behaviour in vivo. In certain cases, the experimental data can be directly translated to in vivo. Sometimes, these data can directly be used as input into physiologically based mechanistic absorption models to predict the drug product behaviour in vivo. Alternatively, the dissolution profiles can be simulated/predicted while using mechanistic or empirical models and used in the physiologically based pharmacokinetic (PBBM) models. Recently, the dissolution data are first being modelled/analysed in vitro and then entered into PBBM models, allowing for better integration of the physiological and biological factors.

As part of cross-work package activities of the Innovative Tools for Oral Biopharmaceutics (OrBiTo) grant supported by the Innovative Medicine Initiative (IMI) (sponsored by the European Union and the European Federation of Pharmaceutical Industries and Associations (EFPIA)), the use of dissolution profiles for incorporation within PBBM models were reviewed. Several grant partners, including pharma, academic, and commercial software developer partners, presented their approaches on the incorporation of the in vitro generated dissolution data to predict the drug product behaviour in vivo. Three specific cases were presented at the meeting that are briefly described here, for further details readers are encouraged to see the relevant references.

Mitra and colleagues generated dissolution profiles in multimedia to support a site transfer for etoricoxib, which is a BCS II drug [28]. The F2 similarity test failed at pH 4.5 and 6.8. They used the in vitro dissolution data into a PBBM model applying the Z-factor approach to assess the potential in vivo impact of the dissolution dissimilarity at those specific pH values. The predicted AUC_0-120_ hr and C_max_ values from the virtual simulation of 120 mg of etoricoxib tables from the two sites formulations were shown to be bioequivalent. The statistical analysis of the bioequivalence (BE) clinical study also showed the formulations from the two sites were also bioequivalent, confirming the outcome of the modelling exercise.

Guiastrennec and co-workers used the Non-Linear Mixed Effect (NLME) approach to model in vitro and in vivo behaviour of an extended release (ER) formulation of Hydroxypropyl Methylcellulose (HPMC) matrix tablet erosion under fasting and postprandial status [29]. They first analysed and modelled the effects of various factors, including formulation composition, media pH, mechanical stress, and the ionic strength on the tables release profiles. This analysis resulted in an empirical model that was capable of simulating the erosion profile in vitro accounting for various factors. The in vitro model was then combined with data that were gathered and analysed from Magnetic Mark Monitoring (MMM) studies to link the in vitro and in vivo conditions. They then applied the gastrointestinal fed conditions to model the prandial effects on the formulation erosion, e.g., pH and mechanical stress to the in vitro model to predict the formulation behaviour in vivo after food.

In the third case study, Pathak and colleagues used an integrated in vitro in vivo extrapolation (IVIVE) approach to predict ketoconazole (KTZ) behaviour in vivo while using in vitro dissolution profiles [30]. The authors presented a stepwise modelling approach where relevant biopharmaceutics parameters for KTZ were determined and/or confirmed from the modelling of in vitro experiments before being directly used within the PBBM model. They used a Bayesian framework where prior information from several experiments were collated and then integrated within the model. The in vitro experiments modelled are: (a) aqueous solubility profiles to determine intrinsic solubility, salt limiting factors to verify pKa; (b) biorelevant solubility measurements to estimate bile-micelle partition coefficients; (c) pH 1.6 Fasted State Simulated Gastric Fluid (FaSSGF) dissolution for formulation disintegration profiling; and, (d) transfer experiments to estimate the supersaturation and precipitation parameters. These parameters were then used within a PBBM model to predict the dissolved and total concentrations of KTZ in the duodenum of a virtual population. Subsequently, the simulated and observed clinical data were successfully compared. This approach facilitates using the potential extrapolation capabilities of the PBBM models to inform/predict unseen scenarios, e.g., predicting food effects. Further, it can also help in the prioritisation and design of in vitro experiments, eliminating redundant experiments.

It was concluded that there are various approaches to incorporate dissolution data into PBBM models and each approach has its own merits and limitations. Therefore, there is currently not a single approach that satisfies all of our needs. It is essential to always select the suitable approach that is based on the questions that are going to be addressed and bear in mind each of the approach assumptions and utilise the outcomes within the realm of the approach assumptions.

### 2.6. Reflection Paper on the Dissolution Specification for Generic Solid Oral Immediate Release Products with Systemic Action—Evangelos Kotzagiorgis (EMA)

Dissolution testing is one of the key tests used as a part of product control to demonstrate that each batch of product produced performs in the same way as the batches tested in the pivotal clinical studies. This reflection paper aims to define the key terms to help companies as they develop their quality control dissolution methods, bring in the link to clinical relevance, and propose a decision tree to help set the regulatory (proposed) dissolution specification.

The focus of the reflection paper is on the in vitro dissolution of orally administered immediate release generic drug products, where the specification should ensure batch-to-batch consistency with the ultimate goal of detecting problems with in vivo bioavailability i.e., discriminate between the BE and non-BE batches. The paper does not address any additional tests that are required in support of biowaiver BE studies.

The highlights from the paper include:

**(1) Key definitions:**

- Biobatch: The batch used in a bioavailability/bioequivalence study or in clinical testing.
- Discriminatory Power: The ability of a test to discriminate between batches with respect to critical process parameters/critical material attributes that may have an impact on bioavailability.
- Dissolution Specification: Quantity (Q) of active substance dissolved in a specified time, expressed as the percentage of product label claim.
- Side batch: The intended lower in vitro release specification derived from the defined manufacturing process by setting the parameters within the range of maximum variability expected from process validation studies.

**(2) Dissolution method development:** the following attributes should be explored and justified, such as: justification on the use of apparatus, surfactant, stirring speed, choice of media based on API physical properties, intended dose range and formulation, sink or non-sink conditions (sink conditions should be obtained but are not mandatory), understanding of method discrimination, and variability. Variability should be investigated, justified, and, if appropriate, addressed by suitable measures.

**(3) Dissolution Method Discriminatory Power:** Ideally, test conditions should enable discrimination between bioequivalent and non-bioequivalent batches. A sufficiently discriminatory dissolution test allows for extrapolation of the result of a bioequivalence study from the bio-batch to commercial batches when a suitable specification of the amount of active substance released at a specific time-point is applied.

The dissolution method development should aim at developing a method that is able to discriminate between batches manufactured with different critical process parameters (CPPs) and/or critical material attributes (CMAs) that may have an impact on the bioavailability. During development, the dissolution results under different test conditions should be compared with the available in vivo pharmacokinetic data to select the most suitable test conditions.

The non-BE batch (or “bad” batch) should have meaningful changes when compared to the applied finished product. Changes can be at the formulation level (quantitative proportions of excipients may be changed, but the excipients should not be completely omitted), CMAs, or slightly modified CPPs. The recommendation is to include this investigation in the dossier so that the Agency can see the evolution.

The paper acknowledges that, for BCS I/III compounds, it might not always be possible to detect any differences in dissolution after meaningful changes and, in these cases, the method might be considered adequate without further justification or may be replaced by a disintegration test.

The reflection paper proposes three approaches in establishing the discriminatory power of the method.

Approach 1: when test product batches with different in vivo behaviour included—the method should be able to discriminate acceptable and non-acceptable batches by setting a suitable specification. Priority should be given to in-vivo discrimination over other factors that influence method selection.

Approach 2: Only batches with acceptable in vivo behaviour included mentioned as “Side Batch” approach—this is applicable when the bioequivalence of a representative batch of test product has been established (PK study 1). If, in addition, “side batches”, representing different in vitro dissolution profiles, derived from the defined manufacturing process by setting process parameters within the range of maximum variability expected from process validation studies have been shown to be bioequivalent versus a representative reference product batch (PK study 2), and then the dissolution profile of such side batches can be used to set a lower specification limit. In this approach, the rank order of the in vivo and in vitro results should be compared and, if it is the same, this might be used as an indicator for suitability of the chosen test conditions.

Approach 3: No batches with different in vivo behaviour included. This approach can be considered for BCS I and III compounds when the BCS biowaiver is applied as per the Bioequivalence Guideline (CPMP/EWP/QWP/1401/98 Rev. 1/Corr **).

**(4) Setting Specifications:** The reflection paper presents a decision tree to increase the consistency in the approach and aid companies in proposing specifications based on the behaviour of the biobatch. The main principle suggested is that the Q value should be set on the basis of the biobatch dissolution result (mean value of 12 units) minus 10% assuming a discriminatory method when in vivo bioequivalence has been established, as described above. The paper recommends the use of specific time-points, i.e., 15 min, 30 min, and 45 min, for setting single point specification.

In conclusion the Reflection Paper does not impose any additional in vivo data or studies, but instead provides a more systematic approach in developing a suitable dissolution method, establishing its discriminatory power and setting appropriate specification that is linked with the in vivo performance. During assessment through pharmaceutical development, the discriminatory power of the chosen dissolution test conditions should be evaluated while considering the available in vivo data; therefore, the selection of the biobatch is important. Pragmatic discrete time-points for setting specification i.e., 15 min, 30 min, and 45 min, as well as Q values of 75%, 80%, and 85% are recommended. The decision tree will help to achieve harmonisation in the evaluation of dissolution specification of generic products and similar principles may be considered for deriving the specification for innovator products, although it is clear that there will be case-by-case decisions for specific drug substances and/or products.

A key step would be to visualise and share the product knowledge in the dossier data so that the Agency can follow the sponsor thinking to ensure that this approach can grow.

### 2.7. Opportunities and Challenges Establishing Clinically Relevant Dissolution Specifications—An Industry Perspective—Andreas Abend, MSD

Developing and justifying dissolution specifications for product release has been, and continue to be, a challenge for both industry and regulatory agencies during the review of market applications for new and generic drugs. In the past, dissolution specifications were established based on batch consistency, however, the emphasis has shifted towards linking in vitro dissolution specifications to in vivo performance. Ideally, this connection is established via in vitro-in vivo correlations; however, this may not always be necessary or practical. The Industry Consortium for Innovation and Quality in Pharmaceutical Development (IQ) developed a roadmap towards establishing Clinically Relevant Dissolution Specifications for Immediate Release solid oral dosage forms in an effort to provide guidance for industry and to encourage productive dialogue with regulatory agencies. The purpose of the roadmap is to describe the best practices towards the development of dissolution specifications that ensure product quality throughout the life cycle of a pharmaceutical product. The roadmap is aligned with QbD principles and it emphasizes the need to identify product performance risks early in the formulation and process development. As such, a thorough understanding of the impact of critical materials attributes and critical process parameters on a product’s in vitro dissolution mechanism as well as potential biopharmaceutics risks is essential when selecting the appropriate conditions (i.e., equipment, media, and agitation). Based on this information, companies might decide to set dissolution specifications via the traditional approach (i.e., based on data generated on pivotal clinical batches), coupled with a robust manufacturing control strategy or invest in dedicated IVIVC studies to link identified CMAs and CPPs to in vivo performance. The latter approach is synonymous of “clinically relevant dissolution specifications” (CRDS), and specific outcomes of these PK studies are usually not predictable. The aspirational goal to establish a Level A IVIVC, is often impossible for IR drugs and Level C correlations or IVIVRs ate typical [11]. The possibility to leverage in silico data to justify in vitro dissolution specifications is gaining a lot of attention and it is an alternative to the traditional IVIVC approach when considering the significant advancements in PBBM modelling and simulations.

For highly soluble rapidly dissolving drugs, pursing the traditional specification setting approach (Option 1 of the CRDS roadmap) is often typically justified; however, for poorly soluble drugs, the risk of establishing a specification that might not detect batches with either poor quality (due to a specification lacking “discriminatory power”), or one that fails production batches with acceptable bioperformance still remains. Companies are encouraged to pursue Option 2 of the roadmap to avoid this dilemma. The need to establish CRDS and the timing of potential IVIVC studies might be informed via the continued biopharmaceutics risk management throughout product development and eventually post-product approval.

Examples of successful application of the proposed CRDS roadmap approaches 1 and 2 were a part of the presentation. Januvia^®^ is a leading oral treatment for Type 2 diabetes mellitus and dissolution specification setting, followed by the traditional approach (approach 1). The drug product is recognized by EMA and FDA as BCS 1 and BCS1/3 respectively. In vitro drug release is mainly driven by API PSD, which is controlled as part of the API release. In the context of the robust manufacturing control strategy, all of the major regulatory agencies accepted MSD’s RTRT approach, which includes at-line disintegration testing as a surrogate for dissolution testing. Furthermore, the US FDA recently accepted at-line hardness testing in lieu of disintegration, since the test provides the same process and product control when compared to disintegration. A second example of approach 1 concerns a fast disintegrating IR tablet containing a BCS 4 compound. The API is blended with excipients, roller compacted, and compressed into tablets. In this case, MSD demonstrated that the in vitro release rate of the drug substance from the tablet is only dependent on release from granules, but not tablet disintegration or the API particle size. Different granules with API having a wide range of PSDs were processed via RC compression and processed into tablets to support the QC dissolution specification. In addition, the API’s intrinsic dissolution, as well as dissolution from the granules, was studied. These studies showed that, while the dissolution method was sensitive towards changes in granule attributes drug, API PSD did not impact the API release. In this case, the specification was set based on clinical batch release data.

BELSOMRA^®^ and GRAZYNA^®^ are two immediate release products containing poorly soluble drug substances, which are processed in amorphous solid dispersions to enhance in vivo drug exposure are examples of product challenges that are associated with over-discriminating dissolution methods. Following the dissolution method development according to guidance resulted in methods for each drug that demonstrated high sensitivity to tablet hardness. As a result of setting specifications based on limited clinical batch experience, the proposed commercial manufacturing process would have required tight process controls and the justification of post-approval changes based on dissolution similarity was considered challenging. IVIVC studies with tablets made under a wide range of compression forces were made and evaluated against tablets made at the target compression force to address this risk. While, for BELSOMRA^®^, a multiple Level C IVIVC was achieved, GRAZYNA^®^ showed no significant PK impact over the study range. In both cases, the results were used to justify wider dissolution specifications and a comfortable process space, thus avoiding the risk of unnecessary product discards.

The acceptance of CRDS by various countries is encouraging, yet world-wide acceptance of clinically relevant drug product specifications, particularly when performed in non-standard dissolution equipment, remains to be seen. Dissolution that is performed following the current regulatory requirements mandate adherence to strict conditions with respect to equipment, media, and amount of drug released. Performing product release testing using non-standard dissolution equipment and media in a Quality Control environment is likely challenging, due to method robustness concerns, even if CRDS established under conditions outside of current guidance were acceptable.

## 3. Roundtable Discussion

The roundtable session provided an opportunity for the delegates to discuss the information shared during the podium presentations, debate key questions, and suggest options to make progress in this critical area of regulatory biopharmaceutics. The major theme of this session was “How should we as Industry and Agencies tackle this together in the future?”. The session was interactive with suggestions and recommendations coming from both Industry members and EU Based regulators. It was clear that there is energy from both parties to move this topic forwards.

Table 2, below, shows the key themes and topics, including the major talking points and take homes or conclusions.

## 4. Meeting Conclusions

The one-day meeting provided an opportunity for Industry and EU Based Regulatory scientists to debate the topic of Clinically Relevant Dissolution Perspectives for Oral Drug products. The meeting heard from both Industrial scientists through case examples of how the thinking and various methodologies can be applied and from regulatory scientists on how dissolution methods are then reviewed, together with the regulatory expectations.

It was clear from the presentations and discussions that, although there has been progress in the field of CRDS from both Industry (wider approach in investment strategies in the setting of clinically relevant dissolution specifications) and from Regulators (approving Clinically Relevant Dissolution Specifications for some products), there is still the need to bring Industry and Regulators closer together. The clear take homes and areas to work on are:

1. Industry partners to increase the communication, strategy sharing (modelling and dissolution method development) and visualisation of drug product development information with regulators during scientific meetings and dossier discussions. Regulatory scientists want to learn together with Industry Scientists.
2. Regulatory scientists to increase collaboration with Industry partners to work further on an EU/EMA framework/roadmap to CRDS, including the key questions in the table above, and build a common understanding of IVIVR modelling approaches and validation (e.g., mechanistic absorption modelling using PBBM). Suggested approaches include a workshop/information day on mechanistic absorption modelling for regulators and cross Industry/Regulatory focussed work groups on the defined topics.
3. Opportunity to engage across Global Regulatory bodies with Industry to determine a harmonised approach for the development of CRDS.

## Figures and Tables

**Table 1:** Comparison of the physico-chemical parameters of JNJ-1 and the newly isolated crystal form.

Starting Material	Solubility (mg/mL)			Intrinsic Dissolution Rate (µg/min/cm^2^)		
	SGF	FaSSIF	FeSSIF	SGF	FaSSIF	FeSSIF
Registered crystal form	0.037	0.54	5.00	3.9	13.8	157.9
New crystal form	0.023	0.31	3.34	2.4	7.3	90.6
**Ratio forms**	**0.62**	**0.57**	**0.67**	**0.62**	**0.53**	**0.57**

**Table 2:** Round Table Discussion: Topics, Major Talking Points and Conclusions/Take Home Messages.

Discussion Topic	Major Talking Points	Conclusions/Take Homes
Big company vs. Biotech approach and funding to CRDS	Agreement	Ideally all Innovators would aim for CRDS
R&D investment with Supply Chain Benefit	Need to increase awareness in R&D on the need for the investment to ensure continued product supply to patients	Companies to increase dialogue and understanding between their own R&D and Supply Chain entities
Approach for all products or approach per therapeutic type?	Ideal would be all products. Timelines, budgets and product volumes and ability to dose healthy volunteers will likely play a role	Industry to set out their approach and strategy and share with Regulators (during dossier discussion)
IVIVC vs. IVIVR approaches and acceptance	For IR products agreement that IVIVC is often impossible. IVIVR (i.e., PBBM) can increase the mechanistic understanding of the drug product	Industry to increase use of PBBM (mechanistic absorption models) in DP understanding and include in CMC sections of dossiers proactively rather than retrospectively
Increase company information sharing with regulators including development investigations—does Industry hold back in case of additional questions and delay in approval?	Companies have the product knowledge. Regulators receive what the companies share and often miss the product development story.	Industry to increase communication and information sharing on new ideas and methodologies to increase regulator buy in and acceptance of new approaches
Availability of relevant data—when and how to collect, how to engage with Clinical folks to include CMC arms in studies	CRDS strategy best discussed after first human safety and Proof of Concept studies where adjustments can still be made to the formulation. CRDS studies best executed post-formulation lock as then highest return on investment	To be successful Industry partners to ensure cross functional/multi-disciplinary dialogue between CMC, Clinical PK, Quality, Modelling & Simulation groups
Companies seem to use PBBM modelling for internal decision making and not proactively in files—why?	Level of model validation, differences in global regulatory acceptance of mechanistic absorption models in specification setting, IVIVC acceptance compared to IVIVR acceptance	Need to increase regulatory awareness, understanding and acceptance of IVIVR/PBBM approaches in the CMC setting. Guidelines on MAM model validation crucial
What do we mean by biorelevant media exactly i.e., recipe?	Clear that there are several meanings in the term biorelevant and biorelevant media	Need agreement on terminology within EMA (see M-CERSI meeting write ups [3,4,31,32])
Dissolution method need to be discriminatory towards CMAs or CPPs which may have an impact on bioavailability. Focus is primarily on BCS 2 and BCS 4 compounds (BCS 1 and 3 through BCS Biowaiver Guidelines).	Key Questions: (1) Is there a need to have a non-BE batch if its outside the formulation and process design space and the originator tests ‘design space variants’ in vivo to set the dissolution spec? (2) Is there an opportunity to discussion on the “minimal dissolution profile” concept to generate the dissolution safe space? (3) Would it be beneficial for originators to discuss their CRS strategy in advance with the agencies to ensure alignment?	Encourage all sponsors to engage as they are putting their CRDS strategy together to discuss e.g., variants to test, dissolution methods under discussion, scenarios to spec setting. Clear that Regulators want to engage with Industry and learn together with Industry.
How do we bring in PBBM modelling to (help) set clinically relevant specs? Focus is primarily on BCS 2/4 compounds (BCS 1 and 3 through Biowaiver Guidelines)	Key Considerations: (1) Clear Intentions on use of the model (2) Requirements on model inputs and level of variability in parameter determination? (3) Which dissolution methods (QC or biorelevant media) are considered appropriate as inputs? (4) Level of detail provided on model parameterization (in house built or commercially available software? (5) Model validation including non-BE batch? What happens if this isn’t available as all DP design space variants overlay and the PBBM model predicts? (6) What constitutes an acceptable prediction error? (7) In which circumstances can we use the models in place of a clinical trial or will the models only be accepted for post-marketing changes?	Beneficial for originators to discuss their modelling strategy in advance with the agencies to ensure alignment. Clear that Industry needs guidance from the regulators on the key considerations noted. Agreement that all the key considerations noted could not be answered at this time, indeed relevant points and required further dialogue e.g., through Industry/Regulatory working groups.

## Abbreviations

API	Active Pharmaceutical Ingredient
APS	Academy of Pharmaceutical Science
BE	Bioequivalent
CHMP	Committee for Medicinal Products for Human Use
CMC	Chemical and Manufacturing Controls
CMA	Critical Material Attributes
CPP	Critical Process Parameters
CRDS	Clinically Relevant Dissolution Specifications
CYP	Cytochrome P450
DP	Drug Product
EMA	European medicines Agency
EU	European Union
FDA	Food and Drug Administration
ICH	International Committee for Harmonisation
IMI OrBiTo	Innovative Medicine Initiative: Innovative Tools for Oral Biopharmaceutics
IR	Immediate Release
IVIVC	In Vitro In Vivo Correlation
IVIVR	In Vitro In Vivo Relationship
MAM	Mechanistic Absorption Modelling
M-CERSI	University of Maryland Center for Excellence in Regulatory Science and Innovation
NDA	New Drug Application
PhEUR	European Pharmacopia
PBPK	Physiology Based Pharmacokinetics
PK	Pharmacokinetics
QbD	Quality by Design
QC	Quality Control
QTPP	Quality Target Product Profile
R&D	Research and Development
SIF	Simulated Intestinal Fluid
SGF	Simulated Gastric Fluid
